# Value of intravenous alteplase before thrombectomy among patients with tandem lesions and emergent carotid artery stenting: A subgroup analysis of the SWIFT DIRECT trial

**DOI:** 10.1111/ene.16256

**Published:** 2024-02-26

**Authors:** Adnan Mujanovic, Tomas Dobrocky, Waltraud Pfeilschifter, Luca Remonda, Jildaz Caroff, Daniel Behme, David J. Seiffge, Carlo W. Cereda, Georg Kägi, Joe Leyon, Eike I. Piechowiak, Vincent Costalat, Judith Wagner, Emmanuel Chabert, Thomas R. Meinel, Olav Jansen, Angelika Alonso, Christian Loehr, David S. Liebeskind, Jan Gralla, Urs Fischer, Johannes Kaesmacher, the SWIFT DIRECT investigators

**Affiliations:** ^1^ Department of Diagnostic and Interventional Neuroradiology, University Hospital Bern Inselspital University of Bern Bern Switzerland; ^2^ Department of Neurology, University Hospital Frankfurt Goethe University Frankfurt am Main Germany; ^3^ Department of Neuroradiology Cantonal Hospital Aarau Aarau Switzerland; ^4^ Department of Interventional Neuroradiology, NEURI Brain Vascular Center, Bicêtre Hospital Paris‐Saclay University Le Kremlin‐Bicêtre France; ^5^ Department for Neuroradiology, Otto von Guericke University Hospital Magdeburg University of Magdeburg Magdeburg Germany; ^6^ Department of Neurology, University Hospital Bern Inselspital University of Bern Bern Switzerland; ^7^ Stroke Center, Neurology, Neurocenter of Southern Switzerland (EOC) Lugano Switzerland; ^8^ Department of Neurology Cantonal Hospital St. Gallen, University of St. Gallen St. Gallen Switzerland; ^9^ Department of Neuroradiology St. George's University Hospital London UK; ^10^ Department of Neuroradiology University Hospital Montpellier Montpellier France; ^11^ Department of Neurology Kepler University Hospital, Johannes Kepler University Linz Linz Austria; ^12^ Department of Neurology, Evangelisches Klinikum Gelsenkirchen Academic Hospital University Essen‐Duisburg Gelsenkirchen Germany; ^13^ Department of Neuroradiology University Hospital Clermont‐Ferrand Clermont‐Ferrand France; ^14^ Department of Radiology and Neuroradiology University Hospital Schleswig‐Holstein, University of Kiel Kiel Germany; ^15^ Department of Neurology, Medical Faculty Mannheim University of Heidelberg Mannheim Germany; ^16^ Department of Radiology and Neuroradiology Klinikum Vest Recklinghausen Germany; ^17^ Department of Neurology and Comprehensive Stroke Center, David Geffen School of Medicine University of California, Los Angeles Los Angeles USA; ^18^ Department of Neurology University Hospital Basel, University of Basel Basel Switzerland

**Keywords:** extracranial stent, intravenous thrombolysis, mechanical thrombectomy, randomized controlled trial, tandem lesion

## Abstract

**Background and purpose:**

The value of intravenous thrombolysis (IVT) in eligible tandem lesion patients undergoing endovascular treatment (EVT) is unknown. We investigated treatment effect heterogeneity of EVT + IVT versus EVT‐only in tandem lesion patients. Additional analyses were performed for patients undergoing emergent internal carotid artery (ICA) stenting.

**Methods:**

SWIFT DIRECT randomized IVT‐eligible patients to either EVT + IVT or EVT‐only. Primary outcome was 90‐day functional independence (modified Rankin Scale score 0–2) after the index event. Secondary endpoints were reperfusion success, 24 h intracranial hemorrhage rate, and 90‐day all‐cause mortality. Interaction models were fitted for all predefined outcomes.

**Results:**

Among 408 included patients, 63 (15.4%) had a tandem lesion and 33 (52.4%) received IVT. In patients with tandem lesions, 20 had undergone emergent ICA stenting (EVT + IVT: 9/33, 27.3%; EVT: 11/30, 36.7%). Tandem lesion did not show treatment effect modification of IVT on rates of functional independence (tandem lesion EVT + IVT vs. EVT: 63.6% vs. 46.7%, non‐tandem lesion EVT + IVT vs. EVT: 65.6% vs. 58.2%; *p* for interaction = 0.77). IVT also did not increase the risk of intracranial hemorrhage  among tandem lesion patients (tandem lesion EVT + IVT vs. EVT: 34.4% vs. 46.7%, non‐tandem lesion EVT + IVT vs. EVT: 33.5% vs. 26.3%; *p* for interaction = 0.15). No heterogeneity was noted for other endpoints (*p* for interaction > 0.05).

**Conclusions:**

No treatment effect heterogeneity of EVT + IVT versus EVT‐only was observed among tandem lesion patients. Administering IVT in patients with anticipated emergent ICA stenting seems safe, and the latter should not be a factor to consider when deciding to administer IVT before EVT.

## INTRODUCTION

Acute ischemic stroke (AIS) patients with tandem lesion are frequently treated with emergent internal carotid artery (ICA) stenting. Observational studies suggested improved clinical outcomes and better reperfusion rates among patients undergoing emergent stenting [1, 2, 3]. However, there is presently no consensus on the value of intravenous thrombolysis (IVT) in tandem lesion patients who are undergoing endovascular treatment (EVT) [4, 5, 6]. More severe perfusion alterations in tandem lesion patients and emergent stenting necessitating antithrombotics may potentially increase the risk of bleeding [4, 5, 6]. This has suggested tandem lesions patients as a potential subgroup of stroke patients benefitting from EVT alone [7].

However, observational studies suggested that ICA stenting is safe in AIS patients pretreated with IVT, and a recent individual patient data analysis of the pooled Thrombectomy In Tandem Lesions (TITAN) and Endovascular Treatment in Ischemic Stroke (ETIS) registries suggested that patients treated with EVT and IVT had better outcomes than those treated with EVT alone, but these data are confounded by indication [8, 9, 10, 11]. In all six randomized controlled trials (RCTs) comparing EVT + IVT versus EVT alone, no treatment effect heterogeneity regarding the presence of a tandem lesion was found, but analysis on subgroups of patients undergoing emergent stenting and reports on technical efficacy outcomes are lacking [12, 13, 14, 15, 16, 17].

Based on the available evidence, we hypothesized that IVT is safe in tandem lesion patients undergoing emergent ICA stenting. To test this, we conducted an exploratory subgroup analysis of the Solitaire With the Intention for Thrombectomy Plus Intravenous t‐PA Versus Direct Solitaire Stent‐Retriever Thrombectomy in Acute Anterior Circulation Stroke (SWIFT DIRECT) RCT.

## METHODS

### SWIFT DIRECT Trial

SWIFT DIRECT was one of the six principle RCTs that investigated effects of IVT in AIS patients directly admitted to an EVT‐capable stroke center by randomizing them to either EVT + IVT or EVT‐only treatment arms (clinicaltrials.gov unique identifier: NCT03192332). Full trial details have been described in the main paper [12]. Briefly, SWIFT DIRECT was an open‐label RCT conducted at 48 stroke centers, in eight countries, with 408 IVT‐eligible patients randomized into two treatment arms with 1:1 allocation [12]. Full inclusion and exclusion criteria are listed in Table S1. The study was approved by all local ethics committees (Central Ethics Committee Bern ID 2017–00974), and reported following CONSORT (Consolidated Standards of Reporting Trials) guidelines.

### Definition and treatment of tandem lesions

In the SWIFT DIRECT trial, tandem lesion was defined as clinically significant atherosclerotic stenosis or occlusion of the extracranial ICA (i.e., ≥90% stenosis) ipsilateral to the intracranial target lesion. Tandem lesion was also a stratification factor for the main trial analysis and was site‐adjudicated at the time of randomization [12]. For the treatment of tandem lesion patients, balloon guide catheter or distal aspiration catheter were used according to the institutional standard method. Antiplatelet medication was administrated by the treating physicians on an individual case basis.

### Primary and secondary endpoints

Primary endpoint of this subanalysis was functional independence at 90 days, defined as a modified Rankin Scale (mRS) score of 0–2. The mRS score was assessed by an independent and blinded rater during the routinely scheduled in‐person clinical visit 90 days after the indexed event or with a structured telephone interview in the case a patient was not able to come to the hospital.

Secondary endpoints of this study were angiographic reperfusion success, symptomatic intracranial hemorrhage rates, and 90‐day all‐cause mortality. Angiographic reperfusion was evaluated on an extended Thrombolysis in Cerebral Infarction (eTICI) scale, where cross‐sectional eTICI (cs‐eTICI) ≥ 2b50 on final angiographic imaging constituted successful reperfusion. Details on postinterventional cs‐eTICI evaluation have already been described in detail [18]. Classification of intracranial hemorrhage was performed according to the Heidelberg Bleeding Classification scale [19]. Symptomatic intracranial hemorrhage (sICH) was noted in the case of an increased neurological deterioration (reflected by a change of 4 or more points on the National Institutes of Health Stroke Scale [NIHSS] scale or the patient's death) together with associated hemorrhage on the 24 h follow‐up imaging. Stent patency was also evaluated on the follow‐up imaging at 24 h. All imaging data were evaluated by an independent core laboratory.

### Statistical analysis

Categorical data are presented using absolute and relative frequencies, continuous data using median and interquartile range (IQR). Crude comparisons were made using Fischer exact and Mann–Whitney–Wilcoxon tests for categorical and continuous variables, respectively. The effect of allocation to EVT + IVT versus EVT‐only according to tandem lesions was analyzed using Firth logistic regression models with allocation, tandem lesion, and their interaction as covariates. Firth logistic regression is a penalized maximum likelihood method that reduces small‐sample bias. Models were adjusted for sex and binary stratification variables: NIHSS at baseline (≤17 vs. >17), age (<70 vs. ≥70 years), location of the initial lesion (first segment of the middle cerebral artery [M1] only versus ICA or ICA and M1 together), and Alberta Stroke Program Early CT Score (ASPECTS; 4–7 vs. 8–10). Marginal odds ratios with 95% confidence intervals (CIs) in each subgroup and *p*‐value for interaction are presented. For sensitivity purposes, we included unadjusted models and conventional maximum likelihood logistic regressions. Probability values were not adjusted for multiplicity and have to be interpreted accordingly. Smaller *p*‐values should be interpreted as more evidence against the null hypothesis, but a significance threshold is not used. Treatment effect heterogeneity refers to different treatment effect in a subpopulation (e.g., a benefit of EVT + IVT in non‐tandem lesion patients but no benefit in patients with a tandem lesion). If statistically significant heterogeneity is shown, this suggests that the treatment effect of the subgroups should inform treatment decisions, rather than the overall effect. However, interpretation of heterogeneity should also take into account other factors, such as the pathophysiological background of the findings, how many subgroups were tested, and if analyses were prespecified [20]. All analyses were performed in Stata v17.0, and figures were created in R v4.0.3.

## RESULTS

### Baseline characteristics

In total, 408 patients were randomized. Median age of the cohort was 72 years (IQR = 64–81), 51.2% were female, and median NIHSS score was 17 (IQR = 13–20). We have identified 63 (15.4%) patients with a tandem lesion, of whom 33 (33/63, 52.4%) were randomized to EVT + IVT and 30 (30/63, 47.6%) to the EVT‐only arm. Among patients with tandem lesions, 20 had undergone emergent ICA stenting (EVT + IVT: 9/33, 27.3%; EVT: 11/30, 36.7%). When comparing tandem and non‐tandem lesion AIS patients, tandem lesion patients were younger (69 vs. 74 years, *p* = 0.05), more often male (73% vs. 44.3%, *p* < 0.001), had higher hemoglobin values (143 vs. 136 g/L, *p* = 0.006), shorter time from randomization to groin puncture (24 vs. 29 min, *p* = 0.03), longer time from groin puncture to reperfusion (60 vs. 30 min, *p* < 0.001) and were more likely to have a distal ICA occlusions (74.5% vs. 19.7%, *p* < 0.001). Other characteristics were comparable between both groups, as seen in Table 1.

**TABLE 1:** Baseline characteristics stratified by the presence of tandem lesion.

Characteristic	Total, *N* = 408, N*	Total	Non‐tandem lesion, *n* = 345, N*	Non‐tandem lesion	Tandem lesion, *n* = 63, N*	Tandem lesion	*p*
Age at inclusion, yr, median (IQR)	408	72 (64–81)	345	74 (64–81)	63	69 (61–76)	0.05
Female sex, *n* (%)	408	209 (51.2%)	345	192 (55.7%)	63	17 (27.0%)	<0.001
NIHSS, median (IQR)	408	17 (13–20)	345	17 (13–20)	63	17 (14–19)	0.87
Prestroke mRS, *n* (%)	408		345		63		
0		346 (84.8%)		292 (84.6%)		54 (85.7%)	0.15
1		61 (15.0%)		53 (15.4%)		8 (12.7%)	
4		1 (0.2%)		0 (0.0%)		1 (1.6%)	
Systolic blood pressure, mmHg, median (IQR)	403	147 (131–162)	341	147 (131–160)	62	149 (134–165)	0.52
Diastolic blood pressure, mmHg, median (IQR)	400	80 (70–90)	338	80 (70–90)	62	82 (75–90)	0.30
Heart rate, beats per minute, median (IQR)	397	74 (64–88)	335	75 (63–87)	62	73 (64–90)	0.92
Risk factors, *n* (%)							
Previous ischemic stroke	394	41 (10.4%)	334	38 (11.4%)	60	3 (5.0%)	0.17
Previous transient ischemic attack	389	21 (5.4%)	330	15 (4.5%)	59	6 (10.2%)	0.11
History of hypertension	398	239 (60.1%)	339	206 (60.8%)	59	33 (55.9%)	0.56
History of atrial fibrillation	387	39 (10.1%)	327	35 (10.7%)	60	4 (6.7%)	0.48
History of hypercholesterolemia	387	131 (33.9%)	328	113 (34.5%)	59	18 (30.5%)	0.65
Previous intracerebral hemorrhage	397	2 (0.5%)	336	1 (0.3%)	61	1 (1.6%)	0.28
Prior myocardial infarction	390	41 (10.5%)	331	34 (10.3%)	59	7 (11.9%)	0.65
Medication, *n* (%)							
Warfarin or other anticoagulant	408	16 (3.9%)	345	14 (4.1%)	63	2 (3.2%)	1.00
Aspirin	408	105 (25.7%)	345	89 (25.8%)	63	16 (25.4%)	1.00
Statin or other lipid‐lowering agent	408	119 (29.2%)	345	104 (30.1%)	63	15 (23.8%)	0.37
Laboratory values, median (IQR)							
Blood glucose level, mmol/L	385	6.5 (5.8–7.5)	324	6.5 (5.8–7.5)	61	6.6 (5.9–7.7)	0.88
INR	320	1.0 (1.0–1.1)	272	1.0 (1.0–1.1)	48	1.0 (1.0–1.1)	0.97
Platelet count × 10 E9, mm^3^	405	226 (189–270)	342	226 (190–273)	63	223 (172–260)	0.14
Hemoglobin, g/L	408	137 (125–147)	345	136 (125–145)	63	143 (131–150)	0.006
Imaging							
Baseline imaging, *n* (%)	408		345		63		0.20
CT		205 (50.2%)		167 (48.4%)		38 (60.3%)	
MRI		200 (49.0%)		175 (50.7%)		25 (39.7%)	
Both		3 (0.7%)		3 (0.9%)		0 (0.0%)	
ASPECTS [core lab], median (IQR)	407	8.0 (7.0–9.0)	344	8.0 (7.0–9.0)	63	8.0 (7.0–9.0)	0.84
Baseline intracranial occlusion site, *n* (%)	408		345		63		
Distal ICA		115 (28.2%)		68 (19.7%)		47 (74.5%)	<0.001
Distal ICA and M1		2 (0.5%)		0 (0.0%)		2 (3.2%)	
Proximal M1		144 (35.3%)		134 (38.8%)		10 (15.9%)	
Distal M1		125 (30.6%)		122 (35.4%)		3 (4.8%)	
Proximal M2		18 (4.4%)		18 (5.2%)		0 (0.0%)	
Distal M2		4 (1.0%)		3 (0.9%)		1 (1.6%)	
Distal occlusion sites, *n* (%)	408		345		63		
No		261 (64.0%)		202 (58.6%)		59 (93.7%)	<0.001
Yes		147 (36.0%)		143 (41.4%)		4 (6.3%)	
Timelines, min, median (IQR)							
Time from stroke onset to randomization	408	129 (100–170)	345	128 (99–165)	63	134 (108–178)	0.25
Time from arrival at emergency department to IVT	207	55 (38–71)	173	56 (38–72)	34	47 (32–62)	0.13
Time from arrival at emergency department to groin puncture	408	78 (62–94)	345	79 (63–95)	63	75 (51–85)	0.06
Time from randomization to groin puncture	408	28 (20–38)	345	29 (21–39)	63	24 (18–35)	0.035
Time from start of IVT to groin puncture	207	24 (15–35)	173	25 (16–36)	34	23 (11–32)	0.26
Time from groin puncture to reperfusion	375	32 (21–50)	316	30 (20–41)	59	60 (35–79)	<0.001

*Note*: N* shows the number of patients with missing data.

Abbreviations: ASPECTS, Alberta Stroke Program Early CT Score; CT, computed tomography; ICA, internal carotid artery; INR, international normalized ratio; IQR, interquartile range; IVT, intravenous thrombolysis; M1, middle cerebral artery; MRI, magnetic resonance imaging; mRS, modified Rankin Scale; NIHSS, National Institutes of Health Stroke Scale.

### Effects of IVT

**FIGURE 1:**
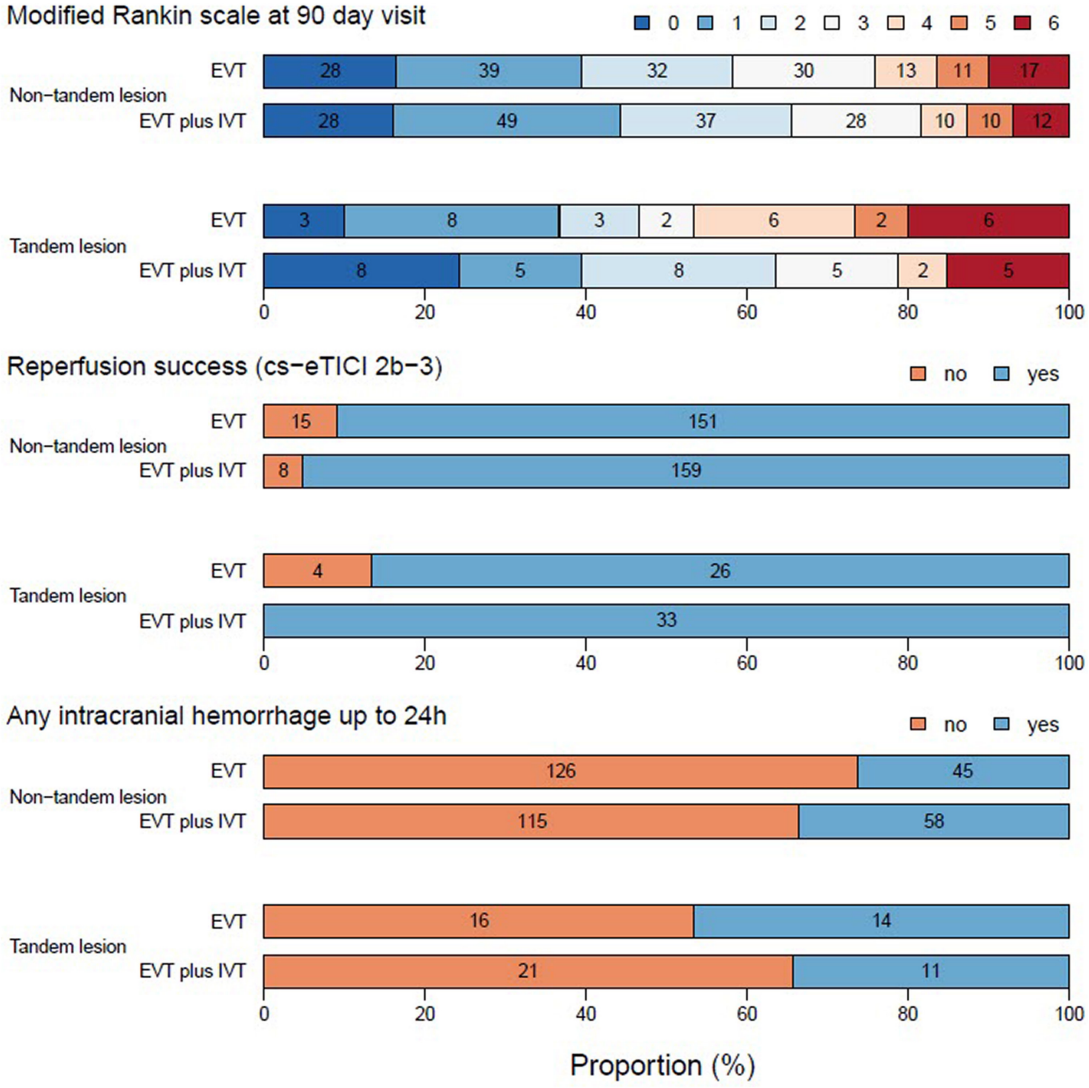
Outcomes stratified on the tandem lesion presence. EVT, endovascular therapy; IVT, intravenous thrombolysis; cs‐eTICI, cross‐sectional extended Thrombolysis in Cerebral Infarction. Functional outcome, evaluated as the shift on the modified Rankin Scale, was comparable between the groups. IVT did not show an association with 90‐day functional independence (odds ratio [OR] = 1.47, 95% confidence interval [CI] = 0.97–2.22). However, IVT increased rates of successful reperfusion (OR = 2.47, 95% CI = 1.07–5.69) and all tandem lesion patients in the EVT + IVT arm (*n* = 33/33, 100%) had successful reperfusion. IVT did not appear to increase the rates of intracranial hemorrhage, as these were comparable between the EVT + IVT and EVT‐only arms (OR = 1.22, 95% CI = 0.80–1.87).

In a crude comparisons between tandem lesion and non‐tandem lesion patients, we did not find evidence for a difference in the primary and secondary outcomes, except for 90‐day mortality, which was higher among tandem lesion patients (17.5% vs. 8.4%, *p* = 0.03; Table 2 and Figure 1).

**TABLE 2:** Primary and secondary outcomes stratified by the presence of tandem lesion.

Outcome	Total, *N* = 408	Non‐tandem lesion, *n* = 345	Tandem lesion, *n* = 63	*p*
Reperfusion success [cs‐eTICI 2b–3], *n* (%)^a^	369 (93.2%)	310 (93.1%)	59 (93.7%)	1.00
Any intracranial hemorrhage up to 24 h, *n* (%)^b^	128 (31.5%)	103 (29.9%)	25 (40.3%)	0.14
ASPECTS at 24 h [core lab], median (IQR)^b^	7.0 (4.0–8.0)	7.0 (4.0–8.0)	7.0 (4.0–8.0)	0.36
Functional independence at 90‐day visit, *n* (%)^c^	248 (60.9%)	213 (61.9%)	35 (55.6%)	0.40
Modified Rankin Scale at 90 day visit, *n* (%)^b^				
0	67 (16.5%)	56 (16.3%)	11 (17.5%)	0.17
1	101 (24.8%)	88 (25.6%)	13 (20.6%)	
2	80 (19.7%)	69 (20.1%)	11 (17.5%)	
3	65 (16.0%)	58 (16.9%)	7 (11.1%)	
4	31 (7.6%)	23 (6.7%)	8 (12.7%)	
5	23 (5.7%)	21 (6.1%)	2 (3.2%)	
6	40 (9.8%)	29 (8.4%)	11 (17.5%)	
Median (IQR)	2.0 (1.0–3.0)	2.0 (1.0–3.0)	2.0 (1.0–4.0)	0.27
Mortality at 90‐day visit, *n* (%)^b^	40 (9.8%)	29 (8.4%)	11 (17.5%)	0.037

Abbreviations: ASPECTS, Alberta Stroke Program Early CT Score; cs‐eTICI, cross‐sectional extended Thrombolysis in Cerebral Infarction; IQR, interquartile range.

^a^ Data missing for 12 patients without a tandem lesion.

^b^ Data missing for one patient with tandem lesion and one patient without a tandem lesion.

^c^ Data missing for one patient without a tandem lesion.

**FIGURE 2:**
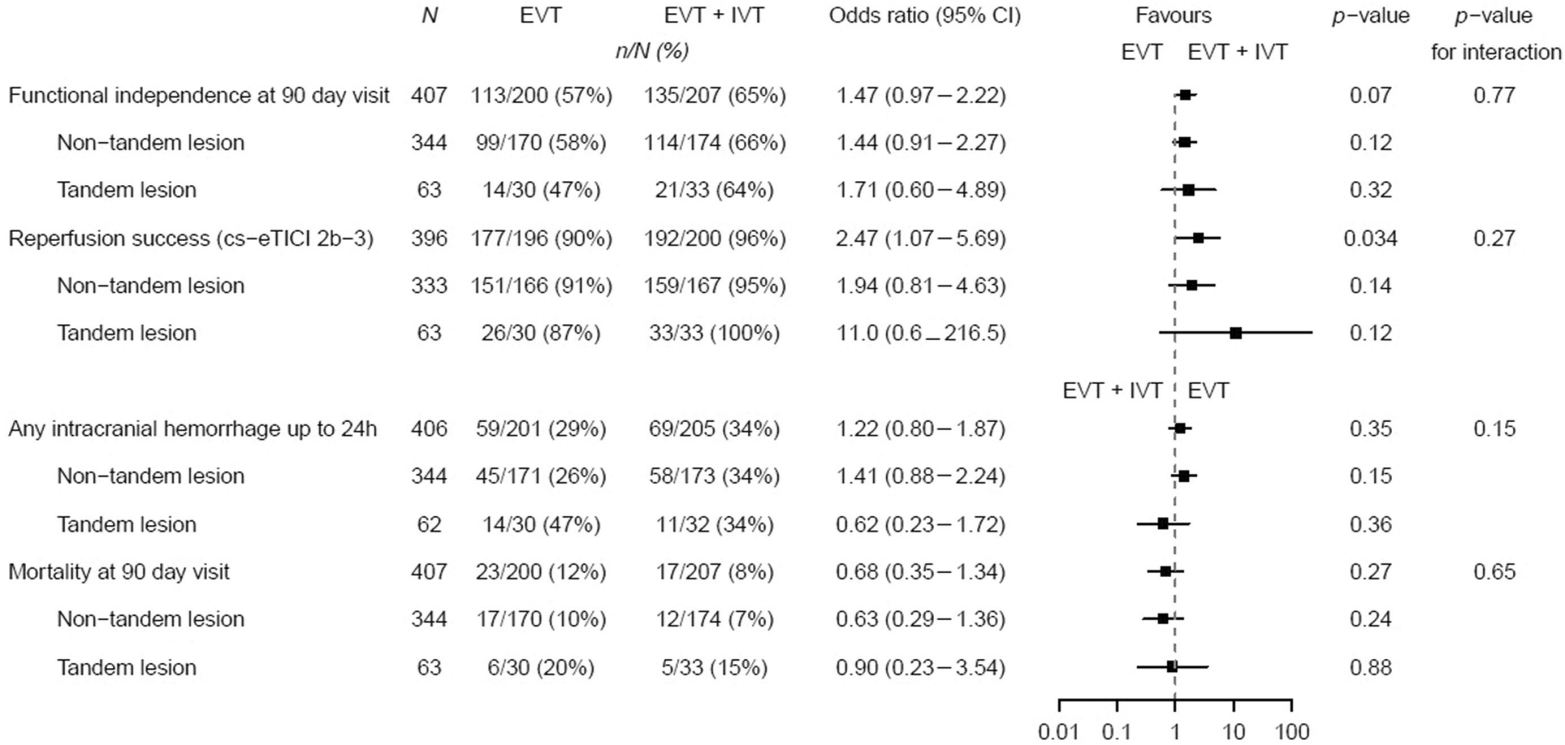
Fitted interaction model of primary and secondary outcomes. Presence of a tandem lesion and emergent stenting of the internal carotid artery did not show treatment effect modification of endovascular treatment (EVT) + intravenous thrombolysis (IVT) versus EVT‐only regarding any predefined clinical and technical efficacy parameters including functional independence at 90 days (*p* for interaction = 0.77), successful reperfusion (*p* for interaction = 0.27), intracranial hemorrhage at 24 h (*p* for interaction = 0.15), and mortality rates at 90 days (*p* for interaction = 0.65). CI, confidence interval; cs‐eTICI, cross‐sectional extended Thrombolysis in Cerebral Infarction.

Rates of successful reperfusion (cs‐eTICI 2b–3) were higher in tandem lesion patients treated with EVT + IVT versus EVT‐only (100% vs. 86.7%, *p* = 0.04), with no evidence of an increased risk of bleeding in the EVT + IVT arm (34.4% vs. 46.7%, *p* = 0.44; Table S2). However, we found no evidence for a treatment effect of EVT + IVT versus EVT‐only on any of the prespecified clinical outcomes. The odds for functional independence, successful reperfusion, intracranial hemorrhage, and mortality were 1.47 (95% CI = 0.97–2.22), 2.47 (95% CI = 1.07–5.69), 1.22 (95% CI = 0.88–1.87), and 0.68 (95% CI = 0.35–1.34), respectively. The presence of a tandem lesion did not show a treatment effect heterogeneity of EVT + IVT versus EVT regarding the occurrence of functional independence at 90 days (*p* for interaction = 0.77; Figure 2), successful reperfusion (*p* for interaction = 0.27), presence of any intracranial hemorrhage at 24 h (*p* for interaction = 0.15), or mortality at 90 days (*p* for interaction = 0.65). An unadjusted Firth model and conventional maximum likelihood models showed comparable results (Figure S1).

### Periprocedural and postrandomization characteristics

Stent patency rates were comparable between the two treatment arms, as all patients who underwent stenting (11/11 patients in the EVT‐only and 9/9 in the EVT + IVT arm) had a patent stent 24 h after randomization. The use of balloon guide catheters in tandem lesion patients was comparable between the treatment arms (EVT‐only vs. EVT + IVT: 13/30 vs. 17/33). Patients with tandem lesion who underwent EVT‐only were more likely to receive periprocedural antiplatelets when compared to patients undergoing EVT + IVT (15/30 vs. 10/33; Table S3). The most common periprocedural antiplatelet medication in both arms was aspirin (EVT‐only vs. EVT + IVT: 13/30 vs. 9/33). Two patients in the EVT‐only arm had received periprocedural aspirin with an additional antiplatelet, and only one patient in the EVT + IVT arm had received a periprocedural antiplatelet different from aspirin. At 24 h after randomization, in the EVT + IVT arm, predominant antiplatelet regimen was either aspirin alone (8/33, 25%) or aspirin with addition of another antiplatelet (8/33, 25%). The latter was also the most common antiplatelet regimen in the EVT‐only arm (8/30, 26.7%). Other periprocedural and postrandomization characteristics were comparable between the groups (Table S3).

## DISCUSSION

This subanalysis of SWIFT DIRECT has the following main findings: (I) In a contemporary multicenter trial comparing EVT + IVT to EVT alone, patients presenting with tandem lesions had similar rates of functional independence, but higher mortality, when compared to patients presenting with an isolated intracranial large vessel occlusion. (II) All tandem lesion patients who had received IVT had successful reperfusion, with no evidence of an increased bleeding risk. (III) Administering IVT was safe in patients with tandem lesions. (IV) There was no treatment effect heterogeneity of EVT + IVT over EVT‐alone according to the presence of a tandem lesion and emergent cervical stenting, implying that these factors should not advise treatment decisions on whether to give or withhold IVT.

### Safety of IVT in tandem lesion patients

Pivotal trials that demonstrated efficacy of IVT over placebo did not report any analysis on specific patient subgroups, such as tandem lesion patients [21], whereas observational studies reported disparate findings [4, 5]. Investigators of the CLOTBUST registry reported that tandem lesion patients had lower reperfusion rates when compared to patients with isolated lesions (9.4% vs. 38.9%, *p* = 0.02) [6].

Meanwhile, EVT had become the standard of care for tandem lesion patients, and recent studies have focused on reporting the efficacy of IVT in the setting of EVT. Investigators of the TITAN registry reported comparable outcomes between EVT‐treated tandem lesion patients with and without IVT and no increased sICH rates (5% vs. 8%, *p* = 0.54) [9]. Pooled analysis of the TITAN and ETIS registries showed higher odds of successful reperfusion in tandem lesion patients with IVT (odds ratio [OR] = 1.1, 95% CI = 1.0–1.2) and again no increase in sICH rates (OR = 0.99, 95% CI = 0.95–1.04). IVT pretreatment seemed effective even in tandem lesion patients who experience ICA dissection (TICI 2b–3 for EVT + IVT vs. EVT‐only: 83% vs. 64%, *p* = 0.02) [11].

Our study confirms prior findings on the association between EVT + IVT and increased reperfusion rates, as all patients with tandem lesion pretreated with IVT experienced successful reperfusion, with no evidence of an increased risk of bleeding. Tandem lesion patients have been suggested as a subgroup of stroke patients who are unlikely to benefit from the IVT treatment approach [7]. This is most likely due to larger clot burden and frequent use of procedural antiplatelets that could potentially increase hemorrhage risks [8, 9]. However, multicenter registry analyses have not reported higher rates of sICH or any bleeding type in tandem lesion patients who have received IVT in addition to EVT [10, 11]. We have also observed no increased hemorrhage risk, providing further evidence on potential safety concerns of IVT in EVT‐treated tandem lesion patients. Potential advantages of additional IVT might include its impact on clot softening and thrombolysis of residual clots in distal arteries, which are not amendable by further mechanical endeavors [7].

### Treatment effect of EVT + IVT over EVT‐alone

Pooled patient‐level data from the HERMES collaboration showed no treatment effect heterogeneity for the presence of tandem lesion (*p* for interaction = 0.17) [22]. Three of five RCTs comparing effects of EVT + IVT over EVT‐alone reported treatment effect based on the presence of tandem lesions [13, 14, 15]. MR CLEAN NO IV, DIRECT‐SAFE, and DIRECT‐MT reported no evidence of treatment effect modification of additional IVT in EVT‐treated tandem lesion patients (OR = 1.9, 95% CI = 0.8–4.2; OR = 1.5, 95% CI = 0.4–5.7; OR = 1.7, 95% CI = 0.7–3.9, respectively) [13, 14, 15]. We also report no treatment effect heterogeneity based on the presence of tandem lesion. This was evident across all predefined clinical and technical efficacy parameters.

The current treatment approach for tandem lesion patients might also include emergent ICA stenting, which has been associated with high rates of successful reperfusion and stent patency at 24 h [23]. However, stenting also requires the use of antiplatelet therapy, which might induce higher bleeding risk, especially in patients undergoing the bridging approach [8, 9, 10, 11]. We could observe hesitancy of nonblinded interventionalists to administer additional antithrombotic agents in the EVT + IVT arm, although we observed no effect of additional IVT on rates of intracranial bleeding.

### Clinical outcome

Evidence on the effect of treatment approach on clinical outcome among patients with tandem lesions is heterogeneous [4, 5, 6, 24, 25, 26]. Previous studies on the IVT‐only approach reported disparate results on clinical outcomes [5, 6]. The authors of one study noted that impact of IVT varied depending on lesion location; tandem lesion patients with distal clot location in the middle cerebral artery had comparable outcomes to patients with isolated intracranial lesions [4]. In regard to the EVT + IVT approach, observational studies showed comparable clinical outcomes at 90 days among patients with tandem lesions and isolated occlusions [24, 25]. Pooled analysis of the TITAN and ETIS registries had shown that the EVT + IVT approach is significantly associated with better clinical outcome among tandem lesion patients (OR = 2.6, 95% CI = 1.4–4.9 for 1‐point improvement in mRS shift analysis) [11]. Analysis of the German Stroke Registry has also reported higher rates of functional outcome in the group with additional IVT (OR = 1.60, 95% CI = 1.1–2.3) [26].

Overall, rates of functional outcome among tandem lesion patients reported here seem comparable to the rates reported in other RCTs and large prospective registries [11, 13, 14, 15, 16, 17, 26], although they had higher mortality rates. This may be because tandem lesion patients had higher rates of occlusions in the distal ICA branch, which have been linked with poorer outcome and larger infarct growth [4, 27]. However, in our cohort, ASPECTS at 24 h were comparable between patients with and without tandem lesion. Other possible explanations are increased atherosclerotic burden and associated comorbidities, but also a technically more challenging approach on the account of additional maneuverers, need for angioplasty or stent placing, longer time delays before randomization and groin puncture, and longer time before achieving successful reperfusion (cs‐eTICI ≥ 2b50). Even though another subanalysis study of SWIFT DIRECT reported no treatment effect modification of time delays on overall mortality, that subanalysis was underpowered to detect treatment effects in highly specified subcohorts, such as tandem lesion patients [28]. However, that subanalysis did report a larger number of tandem lesion patients who had onset to needle puncture of >3 h when compared to <3 h (19.6% vs. 14.2%) [28].

### Limitations

This substudy presents a post‐hoc analysis of an RCT and is therefore is subjected to all the design‐related limitations [29]. Sample size of patients with tandem lesion was modest and not powered to detect a true interaction effect on any of the outcomes; therefore, presented results should be interpreted accordingly. Complete sensitivity analysis among patients who had an emergent ICA stenting‐only was not feasible due to the limited sample size in each treating arm. Administration of antiplatelet medication was on an individual case basis, which hinders the generalizability of present results. Choice of tandem lesion management was at a discretion of the interventionalist, and we did not gather information on which approach (head first vs. neck first) was used; however, we would not expect that to impact any of the outcomes [30].

## CONCLUSIONS

IVT appears to be safe among patients with tandem lesions, leading to higher reperfusion rates without an increase in hemorrhagic risk. Anticipated emergent internal carotid artery stenting should not be a reason to withhold IVT in patients presenting with a thrombectomy‐qualifying intracranial occlusion and direct access to EVT.

## AUTHOR CONTRIBUTIONS

**Adnan Mujanovic:** Conceptualization; investigation; writing – original draft; methodology; visualization; writing – review and editing. **Tomas Dobrocky:** Conceptualization; investigation; writing – review and editing; methodology. **Waltraud Pfeilschifter:** Validation; writing – review and editing; data curation. **Luca Remonda:** Validation; writing – review and editing; data curation. **Jildaz Caroff:** Validation; writing – review and editing; data curation. **Daniel Behme:** Writing – review and editing; validation; data curation. **David J. Seiffge:** Data curation; validation; writing – review and editing. **Carlo W. Cereda:** Validation; writing – review and editing; data curation. **Georg Kägi:** Data curation; validation; writing – review and editing. **Joe Leyon:** Validation; writing – review and editing; data curation. **Eike I. Piechowiak:** Validation; writing – review and editing; data curation. **Vincent Costalat:** Validation; writing – review and editing; data curation. **Judith Wagner:** Validation; writing – review and editing; data curation. **Emmanuel Chabert:** Validation; writing – review and editing; data curation. **Thomas R. Meinel:** Data curation; validation; writing – review and editing. **Olav Jansen:** Validation; writing – review and editing; data curation. **Angelika Alonso:** Data curation; validation; writing – review and editing. **Christian Loehr:** Validation; writing – review and editing; data curation. **David S. Liebeskind:** Validation; writing – review and editing; data curation; resources. **Jan Gralla:** Data curation; resources; writing – review and editing; validation; project administration. **Urs Fischer:** Validation; writing – review and editing; project administration; data curation; resources. **Johannes Kaesmacher:** Data curation; supervision; resources; writing – review and editing; methodology; validation; project administration.

## FUNDING INFORMATION

Medtronic Inc supported the study with an unrestricted grant to the University Hospital Bern. Medtronic Inc had no involvement in the final design, data analysis, or interpretation. University Hospital Bern Inselspital provided additional funding.

## CONFLICT OF INTEREST STATEMENT

T.D. reports paid consultancy work with Microvention outside the submitted work. D.J.S. reports grants from the Swiss National Science Foundation, Bangerter Rhyner Foundation, Swiss Heart Foundation, and AstraZeneca outside this study; and personal fees from Bayer, Alexion, BioXodes, and VarmX outside the submitted work. J.L. reports paid consultancy work with Medtronic, Stryker Neurovascular, and Microvention Europe outside the submitted work. E.I.P. reports grants from the Swiss National Science Foundation. U.F. reports financial support for the present study from Medtronic; research grants from the Medtronic BEYOND SWIFT registry, the Swiss National Science Foundation, and the Swiss Heart Foundation; and consulting fees from Medtronic, Stryker, and CSL Behring (fees paid to institution). J.K. reports financial support from Medtronic for the BEYOND SWIFT registry (fees paid to institution), and research grants from the Swiss National Science Foundation supporting the TECNO trial (fees paid to institution), Swiss Academy of Medical Sciences supporting MRI research (fees paid to institution), and Swiss Heart Foundation supporting cardiac MRI in the etiological workup of stroke patients (fees paid to institution). None of the other authors has any conflict of interest to disclose.

## Supporting information


DATA S1

## Data Availability

Deidentified data and a data dictionary are available from the corresponding author upon reasonable request with a research plan and clearance by the ethics committee. The data are not publicly available due to privacy and ethical restrictions.
