# Supplementary material for: Value of intravenous alteplase before thrombectomy among patients with tandem lesions and emergent carotid artery stenting: A subgroup analysis of the SWIFT DIRECT trial
Source: Eur J Neurol. 2024 Feb 26;31(6):e16256. doi: 10.1111/ene.16256 (PMC11235868; doi:10.1111/ene.16256)
Supplement: Supplementary file 1 — DATA S1 [file ENE-31-e16256-s001.docx]

**SUPPLEMENT MATERIAL**

**Tandem lesions, emergent carotid artery stenting and the value of intravenous alteplase before thrombectomy:**

**a subgroup analysis of the SWIFT DIRECT trial**

**Table of Contents**

- Table S1 Full Inclusion and Exclusion Criteria of the SWIFT DIRECT Trial
- Table S5 Clinical Outcomes by Treatment Group for Patients With Tandem Lesions
- Table S3 Procedural Characteristics in Tandem Lesion Patients
- Figure S1 Sensitivity analysis of Treatment Effect of EVT+IVT vs EVT-only on Primary and Secondary Outcomes According to the Presence of Tandem Lesions.

Table S1 Full Inclusion and Exclusion Criteria of the SWIFT DIRECT Trial

| Inclusion criteria | Exclusion criteria |
| --- | --- |
| Informed consent as documented by signature | Acute intracranial hemorrhage |
| Age ≥ 18 years | Any contraindication for IV t-PA |
| Clinical signs consistent with an acute ischemic stroke | Pre-treatment with IV t-PA |
| Neurological deficit with a National Institutes of Health Stroke Scale score of ≥ 5 and < 30 (deficits judged to be clearly disabling at  presentation) | In-hospital stroke |
| Patient is eligible for IV t-PA | Pregnancy or lactation. A negative pregnancy test before randomization is required for all women with child-bearing potential. |
| Patient is eligible for endovascular thrombectomy | Known (serious) sensitivity to radiographic contrast agents, nickel, titanium metals or their alloys |
| Randomization no later than 4 hours 15 minutes after stroke symptom onset and initiation of IV t-  PA must be started within 4 hours and 30 minutes of stroke symptoms onset (onset time is measured  from the time when the subject was last seen well) | Known current participation in a clinical trial |
| Occlusion (modified treatment in cerebral infarction [mTICI] 0–1) of the intracranial ICA, the M1 segment of the MCA, or both confirmed by computed tomography (CT) or magnetic resonance angiography, accessible for MT | Renal insufficiency as defined by a serum creatinine > 2.0 mg/dl (or 176.8 μmol/l) or glomerular filtration rate (GFR) < 30 mL/min and/or known history of renal insufficiency or requirement for hemodialysis or peritoneal dialysis |
| Core-infarct volume of Alberta Stroke Programme Early CT Score (ASPECTS) greater than or equal to 4 (≥ 4) based on baseline CT or MRI (a region has to have a diffusion abnormality in 20% or more of its volume to be considered MR ASPECTS positive) | Severe comorbid condition with life expectancy less than 90 days at baseline |
|  | Known advanced dementia or significant pre-stroke disability (modified Rankin scale score ≥ 2) |
|  | Foreseeable difficulties in follow-up due to geographic reasons (e.g. patients living abroad) |
|  | Comorbid disease or condition that would confound the neurological and functional evaluations or compromise survival or ability to complete follow-up assessments. |
|  | Subject currently uses or has a recent history of illicit drug(s) or abuses alcohol (defined as  regular or daily consumption of more than four alcoholic drinks per day). Known history of arterial tortuosity, pre-existing stent, other arterial disease and/or known disease  at the femoral access site that would prevent the device from reaching the target vessel and/or  preclude safe recovery after MT |
|  | Radiologically confirmed evidence of mass effect or intracranial tumor (except small meningioma) |
|  | Radiologically confirmed evidence of cerebral vasculitis |
|  | CTA or MRI evidence of carotid dissection |
|  | Evidence of additional distal intracranial vessel occlusion in another territory (i.e. A2 segment of  anterior cerebral artery or M3, M4 segment of MCA) on initial non-contrast computed tomography/MRI or CTA/MRI |

IV t-PA: intravenous tissue-type plasminogen activator; MT: mechanical thrombectomy; ICA: internal carotid artery; MCA: middle cerebral artery; CT: computed tomography; MRI: magnetic resonance angiography; CTA: computed tomography angiography.

Table S2 Clinical Outcomes by Treatment Group for Patients With Tandem Lesions

|  | Total (N = 63) | Total | EVT (N = 30) | EVT | EVT plus IVT (N = 33) | EVT plus IVT | P-value |
| --- | --- | --- | --- | --- | --- | --- | --- |
|  | N* |  | N* |  | N* |  |  |
| Functional independence at 90 day visit - no. (%) | 63 | 35 (55.6%) | 30 | 14 (46.7%) | 33 | 21 (63.6%) | 0.21 |
| Modified Rankin scale at 90 day visit - no. (%) | 63 |  | 30 |  | 33 |  | 0.12 |
| 0 |  | 11 (17.5%) |  | 3 (10.0%) |  | 8 (24.2%) |  |
| 1 |  | 13 (20.6%) |  | 8 (26.7%) |  | 5 (15.2%) |  |
| 2 |  | 11 (17.5%) |  | 3 (10.0%) |  | 8 (24.2%) |  |
| 3 |  | 7 (11.1%) |  | 2 (6.7%) |  | 5 (15.2%) |  |
| 4 |  | 8 (12.7%) |  | 6 (20.0%) |  | 2 (6.1%) |  |
| 5 |  | 2 (3.2%) |  | 2 (6.7%) |  | 0 (0.0%) |  |
| 6 |  | 11 (17.5%) |  | 6 (20.0%) |  | 5 (15.2%) |  |
| median (IQR) | 63 | 2.0 (1.0, 4.0) | 30 | 3.0 (1.0, 5.0) | 33 | 2.0 (1.0, 3.0) | 0.16 |
| Mortality at 90 day visit - no. (%) | 63 | 11 (17.5%) | 30 | 6 (20.0%) | 33 | 5 (15.2%) | 0.74 |
| Reperfusion success (cs-eTICI 2b-3) - no. (%) | 63 | 59 (93.7%) | 30 | 26 (86.7%) | 33 | 33 (100.0%) | 0.046 |
| Any intracranial hemorrhage up to 24h - no. (%) | 62 | 25 (40.3%) | 30 | 14 (46.7%) | 32 | 11 (34.4%) | 0.44 |

N*: number of patients with non-missing data

EVT: endovascular therapy; IVT: intravenous thrombolysis.

Table S3 Procedural Characteristics in Tandem Lesion Patients

|  | Total (N = 63) | Total | EVT (N = 30) | EVT | EVT plus IVT (N = 33) | EVT plus IVT |
| --- | --- | --- | --- | --- | --- | --- |
| **Procedural characteristics** | N* |  | N* |  | N* |  |
| Number of passes - median (IQR) | 63 | 2.0 (1.0, 3.0) | 30 | 2.0 (1.0, 3.0) | 33 | 2.0 (1.0, 3.0) |
| Balloon guide catheter used - no. (%) | 63 | 30 (47.6%) | 30 | 13 (43.3%) | 33 | 17 (51.5%) |
| Conscious sedation - no. (%) | 63 | 29 (46.0%) | 30 | 14 (46.7%) | 33 | 15 (45.5%) |
| General anesthesia - no. (%) | 63 | 27 (42.9%) | 30 | 13 (43.3%) | 33 | 14 (42.4%) |
| Reason for general anesthesia - no. (%) | 27 |  | 13 |  | 14 |  |
| Hospital standard practice |  | 19 (70.4%) |  | 10 (76.9%) |  | 9 (64.3%) |
| Clinically indicated |  | 8 (29.6%) |  | 3 (23.1%) |  | 5 (35.7%) |
| Procedural complications - no. (%) | 63 | 5 (7.9%) | 30 | 3 (10.0%) | 33 | 2 (6.1%) |
| New territory emboli following MT - no. (%) | 63 | 5 (7.9%) | 30 | 3 (10.0%) | 33 | 2 (6.1%) |
| Angioplasty/Stenting (core lab) - no. (%) | 63 | 35 (55.5%) | 30 | 15 (50.0%) | 33 | 20 (60.6%) |
| Procedural antiplatelet - no. (%) | 63 |  | 30 |  | 33 |  |
| None |  | 38 (60.3%) |  | 15 (50.0%) |  | 23 (69.7%) |
| Aspirin alone |  | 23 (36.5%) |  | 13 (43.4%) |  | 9 (27.3%) |
| Other antiplatelet alone |  |  |  | 0 (0.0%) |  | 1 (3.0%) |
| Aspirin and Clopidogrel |  | 1 (1.6%) |  | 1 (3.3%) |  | 0 (0.0%) |
| Aspirin and Ticagrelor |  | 1 (1.6%) |  | 1 (3.3%) |  | 0 (0.0%) |
| **24h post randomization** |  |  |  |  |  |  |
| Stent patency (core lab) - no. (%) | 45 |  | 23 |  | 22 |  |
| Extracranial stent patent |  | 18 (40.0%) |  | 9 (39.1%) |  | 9 (40.9%) |
| Intracranial stent patent |  |  |  | 2 (8.75) |  | 0 (0.0%) |
| No stenting done |  |  |  | 12 (52.2%) |  | 13 (59.1%) |
| Antiplatelet at 24h postrandomization - no. (%) |  |  | 30 |  | 32 |  |
| None |  |  |  | 9 (30.0%) |  | 7 (21.9%) |
| Aspirin alone |  |  |  | 3 (10.0%) |  | 8 (25.0%) |
| Warfarin |  |  |  | 2 (6.7%) |  | 3 (9.4%) |
| Other antiplatelet alone |  |  |  | 1 (3.3%) |  | 0 (0.0%) |
| Aspirin and warfarin |  |  |  | 3 (10.0%) |  | 5 (15.6%) |
| Aspirin and other antiplatelet |  |  |  | 8 (26.7%) |  | 8 (25.0%) |
| Warfarin and other antiplatelet |  |  |  | 2 (6.7%) |  | 0 (0.0%) |
| Aspirin and warfarin and other antiplatelet |  |  |  | 2 (6.7%) |  | 1 (3.1%) |

N*: number of patients with non-missing data

EVT: endovascular therapy; IVT: intravenous thrombolysis.

Figure S1 Sensitivity analysis of Treatment Effect of EVT+IVT vs EVT-only on Primary and Secondary Outcomes According to the Presence of Tandem Lesions.


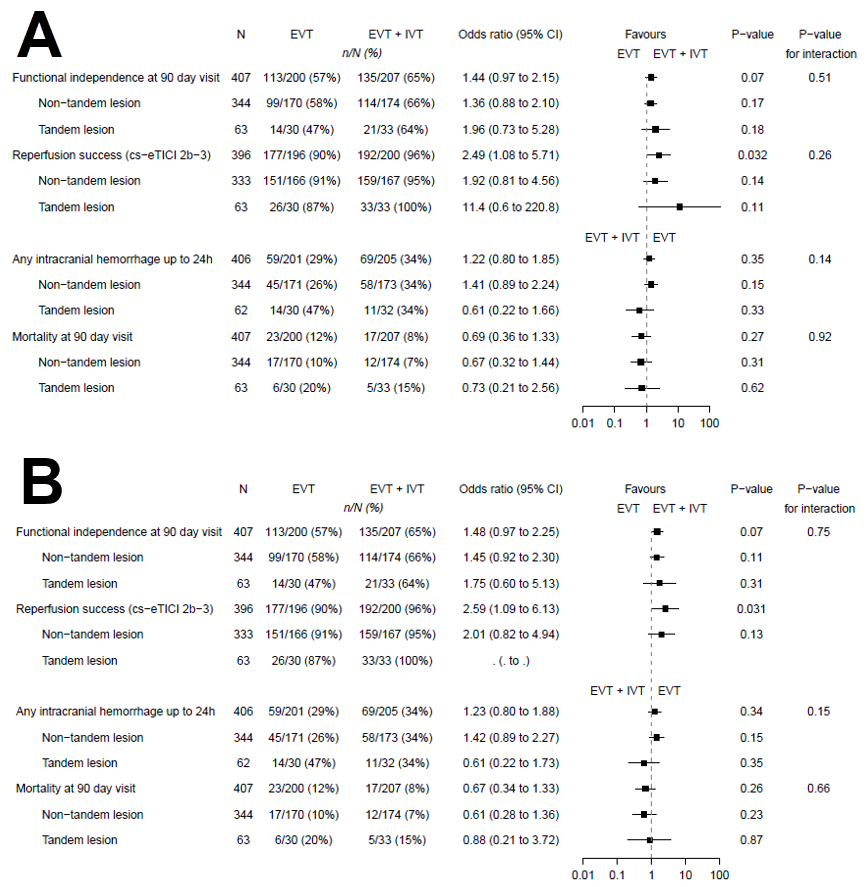


(A) Unadjusted Firth logistic regressions and (B) Conventional maximum likelihood logistic regressions adjusted for stratification factors and sex (see Methods).
